# Proteomic Analysis of a Poplar Cell Suspension Culture Suggests a Major Role of Protein *S*-Acylation in Diverse Cellular Processes

**DOI:** 10.3389/fpls.2016.00477

**Published:** 2016-04-12

**Authors:** Vaibhav Srivastava, Joseph R. Weber, Erik Malm, Bruce W. Fouke, Vincent Bulone

**Affiliations:** ^1^Division of Glycoscience, School of Biotechnology, Royal Institute of Technology, AlbaNova University CentreStockholm, Sweden; ^2^Roy J. Carver Biotechnology Centre, Institute for Genomic Biology, University of Illinois Urbana–ChampaignUrbana, IL, USA; ^3^ARC Centre of Excellence in Plant Cell Walls and School of Agriculture, Food and Wine, The University of Adelaide, Waite CampusUrrbrae, SA, Australia

**Keywords:** poplar, post-translational modification, *S*-acylation, palmitoylation, mass spectrometry, spectral counting

## Abstract

*S*-acylation is a reversible post-translational modification of proteins known to be involved in membrane targeting, subcellular trafficking, and the determination of a great variety of functional properties of proteins. The aim of this work was to identify *S*-acylated proteins in poplar. The use of an acyl-biotin exchange method and mass spectrometry allowed the identification of around 450 *S*-acylated proteins, which were subdivided into three major groups of proteins involved in transport, signal transduction, and response to stress, respectively. The largest group of *S*-acylated proteins was the protein kinase superfamily. Soluble *N*-ethylmaleimide-sensitive factor-activating protein receptors, band 7 family proteins and tetraspanins, all primarily related to intracellular trafficking, were also identified. In addition, cell wall related proteins, including cellulose synthases and other glucan synthases, were found to be *S*-acylated. Twenty four of the identified *S*-acylated proteins were also enriched in detergent-resistant membrane microdomains, suggesting *S*-acylation plays a key role in the localization of proteins to specialized plasma membrane subdomains. This dataset promises to enhance our current understanding of the various functions of *S*-acylated proteins in plants.

## Introduction

The term *S*-acylation encapsulates the chemical linking of two molecules through the formation of a thioester bond (Conibear and Davis, 2010). Some proteins are post-translationally *S*-acylated by fatty acids on specific cysteine residues. The most common type of *S*-acylation is palmitoylation (Wan et al., 2007; Conibear and Davis, 2010; Salaun et al., 2010). This modification involves palmitic acid and promotes the attachment of proteins to biological membranes by increasing their hydrophobicity (Conibear and Davis, 2010). Protein *S*-acylation plays an important role in vesicle trafficking, protein sorting, the formation of protein complexes, and protein partitioning into membrane microdomains (Greaves and Chamberlain, 2007; Levental et al., 2010; Yang et al., 2010; Aicart-Ramos et al., 2011). In addition, and as opposed to other lipid modifications such as myristoylation and prenylation, *S*-acylation is reversible and most often involved in the regulation of the activity of proteins (Hemsley and Grierson, 2008; Blaskovic et al., 2013). *S*-acylation is catalyzed by PATs, known to contain DHHC-CRD required for activity (Lobo et al., 2002; Roth et al., 2002), whereas the reverse reaction is catalyzed by acyl protein thioesterases (APT; Mumby, 1997). The first PATs were identified in the yeast *Saccharomyces cerevisiae* (Lobo et al., 2002; Roth et al., 2002). Following this discovery, several DHHC-CRD containing proteins have been reported in other species (Huang et al., 2004; Hemsley and Grierson, 2008; Ohno et al., 2012). There are seven genes encoding PAT isoforms in the *S. cerevisiae* genome (Roth et al., 2006) whereas the human (Ohno et al., 2012) and *Arabidopsis thaliana* (Hemsley and Grierson, 2008) genomes each contain 24 different *pat* genes. PATs are integral membrane proteins that contain up to six predicted TMDs (González et al., 2011). Only a few plant PATs have been characterized, TIP GROWTH DEFECTIVE 1 (TIP1; Hemsley et al., 2005), PAT10 (Zhou et al., 2013), PAT13 and PAT14 (Lai et al., 2015; Li et al., 2015; Zhao et al., 2016). In *Arabidopsis*, mutations in TIP1 disrupt cell growth as well as the polar growth of root hairs (Hemsley et al., 2005) whereas PAT10 mutants exhibit pleiotropic growth defects and a hypersensitivity to salt stress (Zhou et al., 2013). PAT13 and PAT14 have both been shown to be involved in leaf senescence control (Lai et al., 2015; Li et al., 2015; Zhao et al., 2016).

A large scale proteomic study has led to the identification of around 600 *S*-acylated proteins in *Arabidopsis* cell cultures (Hemsley et al., 2013). However, to date this class of proteins has been studied more thoroughly in humans and yeast than in plants (Martin and Cravatt, 2009; Yang et al., 2010; Martin et al., 2011). Comparatively, our understanding of the function of protein *S*-acylation in plants is limited to a rather small number of examples. For instance, it has been shown in *Arabidopsis* that a Rho-related GTPase is transiently *S*-acylated upon GTP binding and activation, which induces its partitioning into detergent-resistant membranes (Sorek et al., 2007). Resistance of rice against the blast fungus is dependent on the *S*-acylation, subsequent localization in the membrane and activation of a small specific GTPase protein (Kawano et al., 2014). Membrane association of CBLs was also shown to be dependent on *S*-acylation by PAT10 (Zhou et al., 2013). Protein palmitoylation is also known to play a critical role in the polar growth of root hairs in *Arabidopsis* (Zhang et al., 2015). Interestingly, *S*-acylated proteins were found to be largely associated with membrane microdomains in both human and plant cells (Yang et al., 2010; Srivastava et al., 2013), thereby pointing to a general trend for this type of proteins to partition in specialized membrane sub-structures.

As a first step toward the definition of the role of protein *S*-acylation in poplar, we have extended our earlier global proteomic analysis (Srivastava et al., 2013) to the specific identification of *S*-acylated proteins in this plant species. For this purpose, we have combined the ABE method (Drisdel and Green, 2004) with a mass spectrometry based proteomic approach. To date, this is the only study on *S*-acylated proteins in a tree species. In total, we identified around 450 *S*-acylated proteins, the majority of which are involved in signal transduction, response to stress and transport.

## Materials and Methods

### Preparation of Microsomes from Poplar Cell Suspension Cultures

Poplar (*Populus trichocarpa*) cell suspension cultures were generated as previously described (Srivastava et al., 2013) and harvested 7–8 days after inoculation in logarithmic growth phase. Cells from approximately 400 mL of culture were collected on Miracloth (Calbiochem) via vacuum filtration, washed with ice-cold MOPS buffer (50 mM pH 7.0), weighed and re-suspended in 50 mM MOPS buffer (2 mL per gram of fresh weight) containing 5 mM EDTA, 0.33 M sucrose and protease inhibitor cocktail (cOmplete, Mini, EDTA-free, Roche). Cells were disrupted in a Waring blender and the resulting homogenate was centrifuged at 10,000 × *g* for 15 min at 4°C. The supernatant was filtered through Miracloth and spun at 100,000 × *g* for 1 h at 4°C. The microsomal pellet was re-suspended in 2 mL of phosphate buffer saline pH 7.4 (PBS). The Bradford assay was used to measure protein concentration using bovine serum albumin as the standard.

### Purification of *S*-Acylated Proteins

The purification of *S*-acylated proteins was completed using the ABE method, which involves three main steps (Drisdel and Green, 2004; Hemsley et al., 2013). The free cysteine groups are first blocked with *N*-ethylmaleimide (NEM). This is followed by cleavage of *S*-acyl groups by hydroxylamine in the second step. In the final step, the exposed cysteine groups from step 2 are labeled with a thiol specific biotinylation reagent, i.e., Biotin-HPDP (N-[6-(biotinamido) hexyl]-3′-(2′-pyridyldithio) propionamide).

Briefly, 5 mg of protein from the microsomal fraction was incubated overnight at 4°C in 5 mL PBS containing 0.5% SDS, 1% Triton X-100, 0.5% saponin (Calbiochem), protease inhibitor cocktail (cOmplete, Mini, EDTA-free, Roche), 5 mM EDTA and 25 mM NEM (Thermo Scientific). The mixture was spun at 3,500 × *g* for 5 min to remove any insoluble material. Proteins were then precipitated from the supernatant using a chloroform–methanol procedure (Hemsley et al., 2013). The pellet was air-dried and the proteins dissolved in 1 mL of resuspension buffer (PBS containing 8 M urea and 2% SDS). The solution was separated into two aliquots of 500 μL. The first aliquot labeled “(+) hydroxylamine” was mixed with 5 mL PBS containing 0.6 M hydroxylamine (Sigma–Aldrich), protease inhibitor cocktail (cOmplete, Mini, EDTA-free, Roche), 5 mM EDTA and 0.4 mM EZ-Link^TM^ HPDP-Biotin (Thermo Scientific) dissolved in dimethylformamide (DMF, Sigma–Aldrich). The second aliquot labeled, “(-) hydroxylamine” was prepared similarly but without hydroxylamine. Both “+” and “-” hydroxylamine samples were incubated at room temperature (RT) for 1 h on a rotary shaker. Proteins in both samples were then precipitated as above and subsequently dissolved in 4 mL PBS containing 0.1% Triton X-100 and protease inhibitor cocktail (cOmplete Mini, EDTA-free, Roche). This solution was then centrifuged at 9,000 × g at RT for 10 min in order to remove particulates. The supernatant was added to 200 μL of High Capacity Neutravidin^®^ Agarose Resin (Thermo Scientific) which had been previously equilibrated with 0.1% Triton X-100 in PBS for 30 min. This mixture was incubated for 1 h at RT. The beads were recovered on 10 mL disposable columns (Thermo Scientific) by centrifugation at 3,000 × g for 2 min. The beads were then washed three times for 5 min with 5 mL PBS containing 0.2% SDS, 0.2% Triton X-100, and 500 mM NaCl. *S*-acylated proteins were then eluted twice with 500 μL of elution buffer (PBS containing 0.1% SDS, 0.1% Triton X-100, and 1% 2-mercaptoethanol) preheated at 60°C. Proteins in the eluent were precipitated as described above.

In total, three independent biological replicates (BR), each with control (-Hyd) and experimental (+Hyd) samples, were prepared for the identification of *S*-acylated proteins in *Populus*. The samples were designated -Hyd_1, +Hyd_1, -Hyd_2, +Hyd_2, -Hyd_3 and +Hyd_3.

### Protein Separation, In-gel Tryptic Digestion, and Mass Spectrometry

The protein precipitate was resuspended in SDS-buffer [75 mM Tris-HCl buffer pH 6.8 containing 3% (w/v) SDS, 100 mM DTT, 15% (w/v) glycerol and 0.002% bromophenol blue] and proteins were separated on a 10% SDS-PAGE gel and stained overnight using Coomassie Blue (Thermo Scientific, Rockford, IL, USA). Each lane was cut into 14 bands for in-gel proteolysis with trypsin. Each gel piece was incubated in 50 mM ammonium carbonate pH 10 containing 50% acetonitrile. After 1 h at 37°C the liquid was discarded and the gel pieces were dehydrated for 5 min in 50 mM ammonium bicarbonate and 50% acetonitrile. This solution was discarded and the gel pieces were dehydrated again with 100% acetonitrile for 5 min and dried under vacuum. The gel pieces were rehydrated on ice for 15 min in 50 mM ammonium bicarbonate containing 10 ng/μl trypsin and 0.01% Protease MAX (Promega, Madison, WI, USA). The excess of trypsin solution was then removed and the gel pieces were submerged in 50 mM ammonium bicarbonate containing 0.01% ProteaseMAX^TM^ and incubated at 50°C for 2 h. After incubation, the samples were thoroughly agitated (Vortex), centrifuged and the preparations were transferred to new LoBind tubes (Eppendorf). The extracted peptides in each tube were incubated with 0.5% TFA at 37°C for 15 min. The tubes were centrifuged at 16,000 × *g* for 10 min to remove any particulate material and the degraded ProteaseMAX^TM^ surfactant. The supernatants containing peptides were then transferred to fresh tubes, dried and the peptides were dissolved in 0.1% formic acid for mass spectrometric analysis. Peptide analysis was completed using a nanoACQUITY Ultra Performance Liquid Chromatography system coupled to a Q-TOF mass spectrometer (Xevo Q-TOF, Waters, Milford, MA, USA) as previously described (Srivastava et al., 2013).

### Database Searches and Spectral Counting

Our recently implemented Automated Proteomics Pipeline (APP; Malm et al., 2014) was used to analyze mass spectrometry data. APP automates the processing of proteomic tasks including peptide identification, validation, and quantification from LC–MS/MS data and allows for the easy integration of numerous individual proteomic tools (Malm et al., 2014). Briefly, the raw data files were first processed using Mascot Distiller (version 2.4.3.2, Matrix Science, London, UK). The resulting mgf files were converted to the mzML file format using msconvert (Proteowizard) and searched using MS-GF+ (Kim et al., 2010), MyriMatch (Tabb, 2007), Comet (Eng et al., 2013) and X!Tandem (Craig and Beavis, 2004) and the *Populus* protein database (Populus version 3.0; 73,013 entries) with the following settings: trypsin specific digestion with two missed cleavages allowed, peptide tolerance of 100 ppm, fragment tolerance of 0.2 Da, oxidized Met, propionamide and NEM on Cys as variable modifications. The results from all search engines were validated using PeptideProphet (Keller et al., 2002). iProphet (Shteynberg et al., 2011) was used to combine all pep.xml files obtained from PeptideProphet and a protein list was subsequently assembled using ProteinProphet (Nesvizhskii et al., 2003). A concatenated target-decoy database-search strategy showed false positive rates of less than 1% in all searches.

For each protein, peptide sequences were exported with a protein and peptide probability cutoff of 0.95. Peptides matching two or more proteins (shared peptides) were excluded from the analysis. Proteins with no unique peptides (i.e., identified by shared peptides only) were likewise excluded. A protein was considered identified if it contained at least one unique peptide. For each BR, the final protein list obtained after the ProteinProphet step was submitted to an in-house plugin for spectral counting (Zhu et al., 2010). Spectra matching the following criteria were extracted. Unique peptide sequences were filtered out for each protein, with a protein cutoff of 0.95 and a peptide probability cutoff of 0.95. For these peptides, all matching spectra above 0.5 probabilities were indexed for spectral counting (Zhu et al., 2010). Spectral counts were normalized to the total number of spectra counted. The spectral count ratio was calculated only in the case of proteins identified in both +Hyd and -Hyd samples.

### Selection of *S*-Acylated Proteins

For each BR, proteins identified only in +Hyd samples and those exhibiting normalized spectral count ratios >4 (+Hyd/-Hyd) were selected (816 proteins). From this selected group, proteins identified only in one BR +Hyd sample by one unique peptide were removed (298 proteins, Supplementary Table 1). Furthermore, ribosomal proteins as well as proteins which contain thioester linkages not involved in *S*-acylation were also excluded (69 proteins, Supplementary Table 1; Roth et al., 2006; Hemsley et al., 2013). The remaining list was subdivided into two *S*-acylated protein groups. The high probability group (292 proteins) contains proteins identified in +Hyd samples (274 proteins) or proteins whose spectral count ratios were >4 (+Hyd/-Hyd; 18 proteins) in at least two out of three BRs (Supplementary Table 1). The second, medium probability group (157 proteins), comprises proteins identified in +Hyd samples in one BR by two or more unique peptides or in two BR by one unique peptide (Supplementary Table 1).

### Prediction of Topology, Post-translational Modifications, and Other Bioinformatics Analyses

The sequences of all proteins identified as *S*-acylated were used to predict TMDs, myristoylation and *S*-acylation sites. The number of TMDs was determined using the HMMTOP program. *N*-terminal myristoylation and *S*-acylation sites were predicted using the Plant-Specific Myristoylation Predictor and CSS-Palm 4.0 (with high threshold) algorithms, respectively. The iTAK program^1^ was used to classify of all protein kinases experimentally identified as *S*-acylated. Gene ontology information was retrieved from TAIR. The phylogenetic tree was constructed using the ClustalX (version 2.1, Larkin et al., 2007) and TreeView (1.6.6^2^) programs.

## Results and Discussion

**FIGURE 1:**
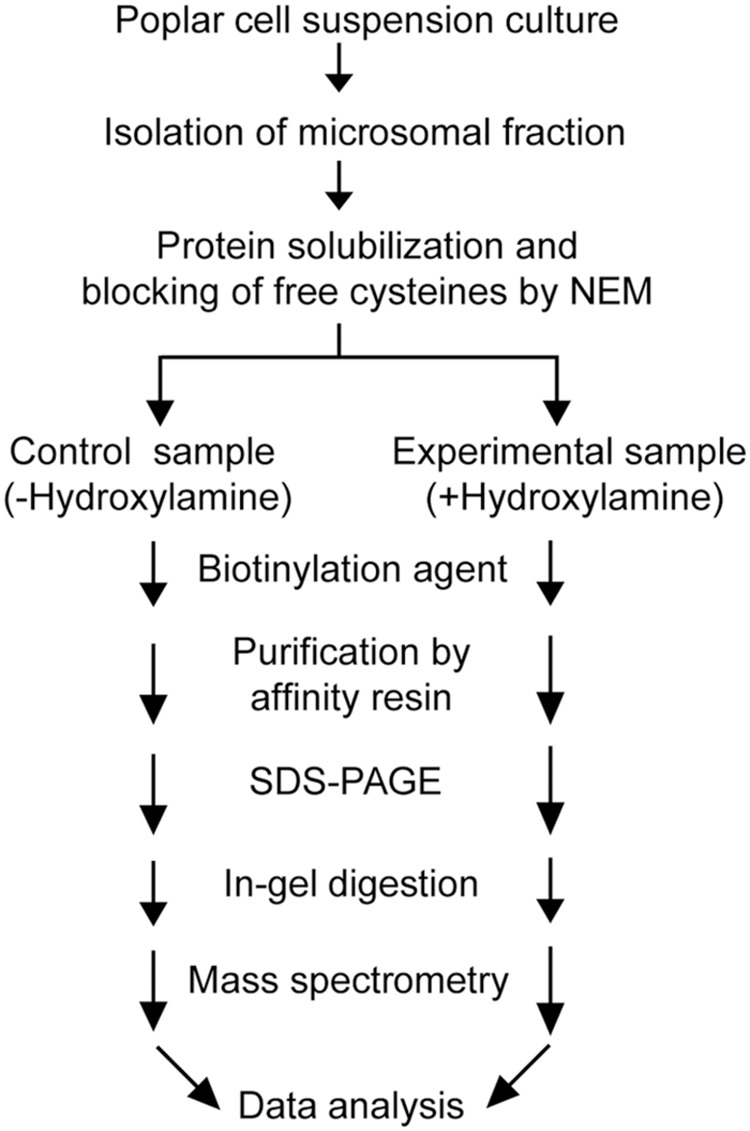
**Experimental set-up used for the purification and identification of *S*-acylated proteins in poplar cell suspension cultures**.

**FIGURE 2:**
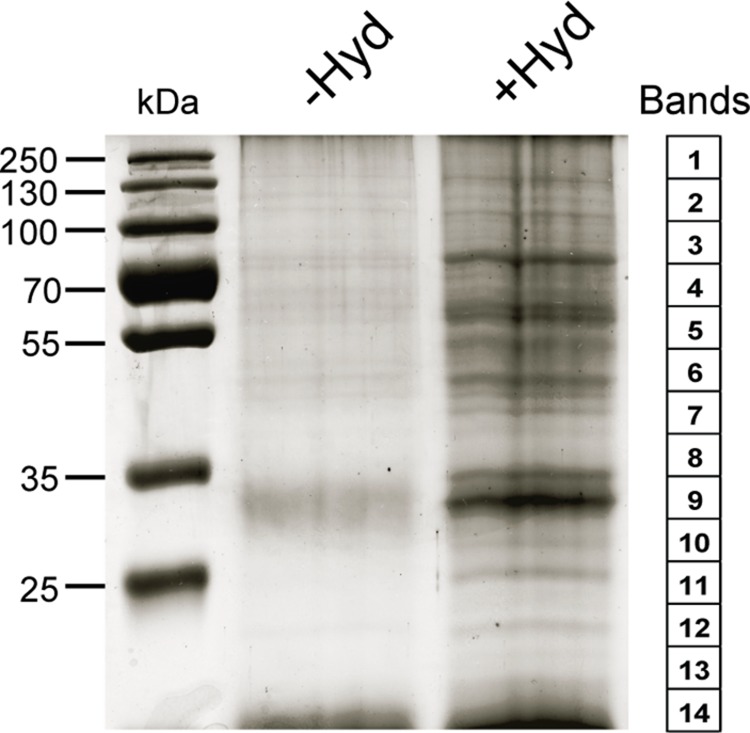
**SDS-PAGE analysis of purified proteins from the control (-Hyd) and experimental (+Hyd) samples**. Each lane of the Coomassie-blue-stained gel was cut into 14 bands as shown on the right side of the picture.

**FIGURE 3:**
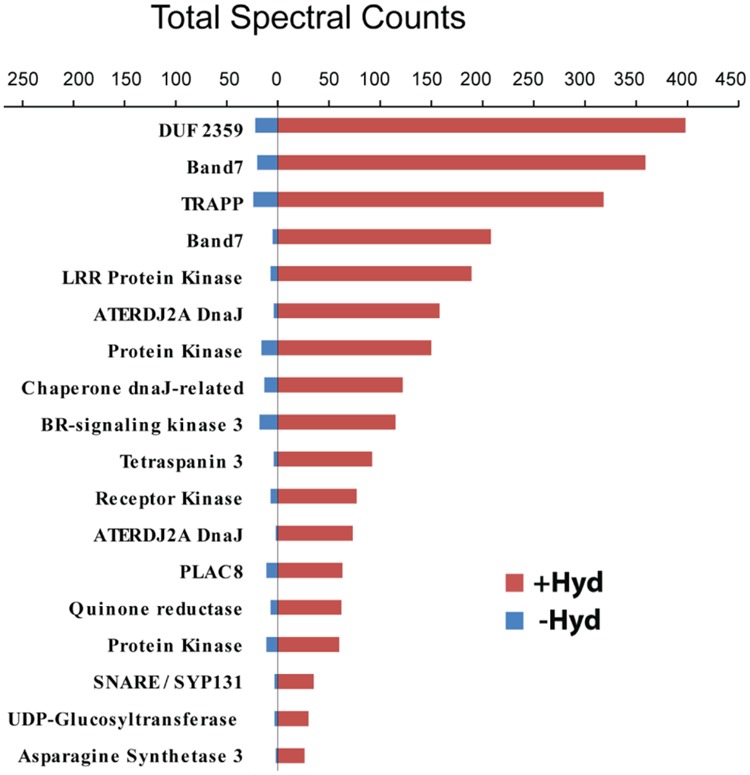
**Comparison of spectral counts for proteins identified in the control (-Hyd) and experimental (+Hyd) samples**.

Post-translational modifications are often essential for the biological activity of proteins and largely contribute to functional diversity (Beltrao et al., 2013). *S*-acylation is a common modification of both membrane and soluble proteins (Conibear and Davis, 2010; Levental et al., 2010; Yang et al., 2010; Aicart-Ramos et al., 2011; Blaskovic et al., 2013), but our understanding of the role of this class of PTM in plants is not as advanced as in yeast and humans. In order to address this question, we have used a proteomic approach to identify *S*-acylated proteins in poplar, as outlined in **Figure 1**. *S*-acylated proteins were purified from a microsomal fraction prepared from cell suspension cultures using the ABE method (Drisdel and Green, 2004). SDS-PAGE analysis revealed clear differences in protein composition between the “-Hyd” and “+Hyd” samples (**Figure 2**). Most of the proteins present in the “+Hyd” preparation represent purified *S*-acylated proteins. However, bands of similar apparent molecular weights were detected in both samples, yet with a much lower intensity in the “-Hyd” control, reflecting the occurrence of some non-specific binding to the Neutravidin^®^ Agarose Resin during the purification step. To exclude false positives from our further analysis of *S*-acylated proteins, spectral counting was used to compare protein abundance between the “+Hyd” and “-Hyd” samples (**Figure 3**).

A total of around 800 proteins were initially identified as being putatively *S*-acylated. The exclusion of false positives followed by strict filtering criteria resulted in two *S*-acylated protein groups (high and medium probability groups). As our work relies on the specific enrichment and identification of *S*-acylated proteins, we chose to examine in further detail only proteins from the high probability group (292 unique proteins; Supplementary Table 1). However, it should be kept in mind that a proportion of the proteins that segregated to the medium probability group (157 unique proteins) might also be *S*-acylated (Supplementary Table 1). The demonstration of which proteins from this second group are actually *S*-acylated requires additional experiments based on complementary techniques (Roth et al., 2006). Here, ribosomal proteins were excluded from the list of palmitoylated proteins while other proteomic studies have suggested that some these proteins are potentially palmitoylated (Roth et al., 2006; Hemsley et al., 2013). Ninety four percent (274 proteins) of the proteins from the high probability group were detectable only in the +Hyd sample, strongly suggesting that these are indeed *S*-acylated. The remaining 6% (18 proteins) were identified in both the -Hyd control and +Hyd samples (**Figure 3**, Supplementary Table 1). Spectral counting is widely used as a semi-quantitative measurement of protein abundance and defined as the total number of spectra identified for a protein (Zhu et al., 2010). The use of this approach revealed that the 18 proteins present in both samples were highly enriched in the +Hyd preparation, suggesting that they are most likely *S*-acylated (**Figure 3**). Interestingly, the closest *Arabidopsis* orthologs of 125 poplar proteins identified in our study were also shown to be *S*-acylated in a recent proteomics study (Hemsley et al., 2013). A total of 582 *S*-acylated proteins has been reported in *Arabidopsis* (Hemsley et al., 2013). However, out of these, 122 proteins only were classified with high confidence as being *S*-acylated (i.e., proteins identified by three peptides or more). It is worth noting that 52 *Arabidopsis* proteins that were identified with low confidence in this species (proteins identified by one peptide only) were also present and *S*-acylated in our samples from poplar, but the confidence for the poplar orthologs was high (Supplementary Table 1). Similarly, 20 *Arabidopsis* proteins classified with medium confidence (proteins identified by two peptides) were present in the high probability group from poplar (Supplementary Table 1). These observations further support the occurrence of *S*-acylation groups in the poplar proteins.

### Functional Classification and Biochemical Properties of *S*-Acylated Proteins

**FIGURE 4:**
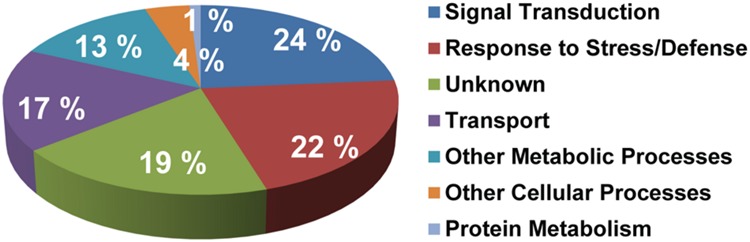
**Functional classification of the high probability group of proteins identified as *S*-acylated.** Gene ontology information was retrieved from TAIR (www.arabidopsis.org).

Functional classification of all *S*-acylated proteins from the high probability group (292 proteins) was completed and resulted in division into a few main categories (**Figure 4**; Supplementary Table 1). Most proteins were principally associated with signal transduction (24%), response to stress (22%) and transport (17%). However, about 17% altogether were related to other metabolic and cellular processes. Many proteins (19%) could not be classified to any functional group. Several proteins in this “unknown” group are either not annotated or have undefined molecular functions (Supplementary Table 1).

Although no consensus sequence for *S*-acylation is known, a few bioinformatic tools are available for the prediction of *S*-acylation sites (Ren et al., 2008). In our study, CSS palm (Ren et al., 2008) was used to search for putative palmitoylated sites in all *S*-acylated proteins identified experimentally. Interestingly, of the 292 proteins examined, 186 were predicted to be *S*-acylated with the remaining 106 containing no such modification (Supplementary Table 1). These data further support the concept that *S*-acylation does not involve a specific common consensus sequence, but instead can be selective depending on the type/group of proteins. It has been proposed that *S*-acylation and *N*-myristoylation can occur simultaneously on some proteins (Batistic et al., 2008). Myristoylation sites were predicted in 41 proteins in our dataset, using the Plant-Specific Myristoylation Predictor algorithm (Supplementary Table 1). This predominately includes receptor kinases, Ca^+2^-dependent protein kinases and other protein kinases, as well as protein phosphatases (Supplementary Table 1). With the exception of 3 proteins, all putatively *N*-myristoylated proteins were also predicted to be *S*-acylated using CSS-PALM (Supplementary Table 1).

Another post-translational lipid modification that often occurs concomitantly with *S*-acylation is prenylation (Aicart-Ramos et al., 2011). Prenylation involves the formation of a thioether bond between a cysteine residue of the target protein and isoprenyl anchors (Aicart-Ramos et al., 2011). It typically mediates protein–membrane and protein–protein interactions (Maurer-Stroh and Eisenhaber, 2005). We used Prenylation Prediction Suite (PrePS; Maurer-Stroh and Eisenhaber, 2005) to search for potential prenylated proteins within our dataset. In the high probability group of 292 *S*-acylated proteins, only six proteins were predicted to be prenylated (Supplementary Table 1). These are a nucleoside triphosphate hydrolase, an alpha/beta-hydrolase, the nucleosome assembly protein NAP1, the heterotrimeric G protein AGG1 and the TOBAMOVIRUS MULTIPLICATION 2A (TOM2a) protein, which is required for efficient multiplication of tobamovirus (Fujisaki et al., 2008; Supplementary Table 1). With the exception of NAP1, these proteins were also predicted to be *S*-acylated using CSS-PALM (Supplementary Table 1). In addition, the AGG1 and TOM2a proteins were experimentally identified as *S*-acylated in *Arabidopsis* (Hemsley et al., 2013). Zeng et al. (2007) also demonstrated that the two heterotrimeric G-protein gamma-subunits from *Arabidopsis*, AGG1 and AGG2, are prenylated. However, another study showed *S*-acylation on AGG2 but not on AGG1, and also suggested that *S*-acylation and prenylation are required for the efficient targeting of these proteins to the plasma membrane (Mason and Botella, 2001).

One function of *S*-acylation is to increase the hydrophobicity of soluble proteins and thus target them to the plasma membrane (Wan et al., 2007; Conibear and Davis, 2010; Salaun et al., 2010). In support of this inference, 130 (out of 292) of the proteins studied have no predicted TMD. In contrast, 97 proteins were predicted to contain one TMD and an additional 65 have two or more putative TMDs (Supplementary Table 1).

### *S*-Acylated Proteins/Protein Families

The *S*-acylated proteins identified in our study are involved in diverse cellular processes (Supplementary Table 1). Interestingly, *S*-acylation was found to occur in four proteins containing DHHC-CRD motifs and known to catalyze the *S*-acylation of other proteins. Thus, it can be concluded that palmitoyl transferases are themselves *S*-acylated (Roth et al., 2006; Hemsley et al., 2013). The most prominent of the *S*-acylated proteins and protein families are discussed below.

#### Protein Kinase Superfamily

**FIGURE 5:**
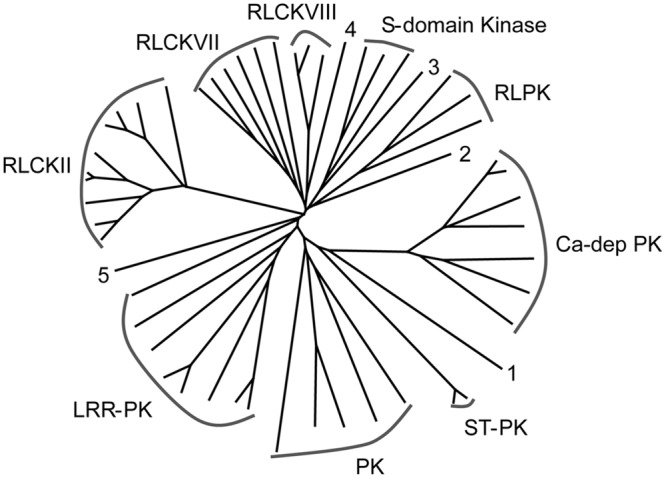
**Phylogenetic tree showing the different classes of *S*-acylated protein kinases identified in the high probability group.** Accession numbers of individual proteins and proteins associated with the different groups are presented in **Table 1**.

The largest group of *S*-acylated proteins identified here is the protein kinase superfamily (**Table 1**; Supplementary Table 1, **Figure 5**). Protein kinases (PKs) are predominantly involved in signal transduction in biological pathways modulating the activity, function, stability, and localization of other proteins via phosphorylation (Torii, 2004; Kemmerling et al., 2011; Lin et al., 2013). It has been established that the main PTM regulating PK activity is phosphorylation (Torii, 2004; Kemmerling et al., 2011; Lin et al., 2013). However, *S*-acylation of many PKs in this and other studies clearly supports the importance of this PTM in regulating PK activity. *S*-acylation may also assist PKs in targeting other proteins for phosphorylation. A total of 52 PKs were identified (**Table 1**; Supplementary Table 1) and information about the classification of PKs into different groups was retrieved from iTAK^3^ Phylogenetic analysis of all identified PKs led to a similar division (**Figure 5**). Thirty-seven proteins identified in our study belong to PK class 1, which contains all transmembrane receptor kinases and related non-transmembrane kinases. The main subfamilies were LRR-RLKs and RLCKs, receptor like proteins (RLPs) and *S*-domain kinases (**Table 1**; Supplementary Table 1, **Figure 5**). Five proteins were present from PK class 2, predominantly including guanylate kinase (GMPK), ATN1-like protein kinase and ankyrin-repeat domain PKs. The conserved cysteine residue(s) typically occurring on the cytoplasmic side of TMDs is considered the site(s) of *S*-acylation in LRR-RLKs (Roth et al., 2006; Hemsley et al., 2013). A mutation in the cysteine residues of flagellin receptor FLS2, a well-known LRR-RLK, abolished *S*-acylation (Roth et al., 2006). The LRR-RLKs identified here also have one or two cysteine residues similarly positioned, suggesting putative *S*-acylation sites (Supplementary Figure 1). Yet another group of proteins found in this category is receptor-like cytoplasmic kinases (RLCKs; 19 proteins). Together with RLKs, RLCKs are involved in transducing diverse signaling pathways to regulate plant growth, development, and immunity (Lin et al., 2013). In *Arabidopsis*, BSK, an RLCK, mediates signal transduction from the receptor kinase BRI1 (Tang et al., 2008). Recently, BSK1 was discovered to physically interact with FLS2 to regulate plant innate immunity (Shi et al., 2013). Many RLCKs lack TMDs and their association with the plasma membrane has been attributed to lipid modifications including *N*-myristoylation and *S*-acylation. Many Ca^+2^-dependent PKs (CDPKs) from class 4 and two PKs from class 5 were also identified as being *S*-acylated in this work (**Table 1**; Supplementary Table 1). CDPKs act as signaling hubs in plant stress signaling and development (see Schulz et al., 2013 and references therein). CDPKs contain a variable N-terminal domain, which is required for membrane association. All *S*-acylated CDPKs identified here also contain putative *N*-myristoylation sites (Supplementary Table 1). Further supporting the role of CDPKs in membrane association, *Arabidopsis* CDPKs variants lacking glycine and/or cysteine required for such lipid modifications are defective in their membrane association (Mehlmer et al., 2010). Furthermore, it was shown that myristoylation and *S*-acylation are both required for localization and full association of rice CDPK (OsCPK2) to membranes (Martin and Busconi, 2000).

**Table 1:** List of *S*-acylated protein kinases identified in the high probability group.

PKs	Class	Accession Number(s)
1	4	Potri.009G012900.1
2	1	Potri.003G032100.1
3	1	Potri.012G055500.1
4	1	Potri.018G081300.1
5	1	Potri.011G045600.1
Ca-dep PK	4	Potri.010G244800.1, Potri.016G117200.1, Potri.011G003400.1, Potri.012G071700.1, Potri.004G207300.1, Potri.009G052700.1, Potri.001G257100.1
LRR-PK	1	Potri.015G035500.1, Potri.012G044600.1, Potri.018G074300.1, Potri.008G144900.1, Potri.010G097200.1, Potri.018G141000.1, Potri.016G126300.1, Potri.014G195100.1,
PK	2	Potri.004G064400.1, Potri.001G343900.1, Potri.012G079000.1, Potri.018G001800.1, Potri.008G156000.1
RLCKII	1	Potri.004G182800.1, Potri.005G249300.1, Potri.002G011800.1, Potri.014G179300.1, Potri.014G082400.1, Potri.001G246400.1, Potri.003G126700.1, Potri.001G104700.1, Potri.016G105800.1, Potri.006G093900.2
RLCKVII	1	Potri.008G205700.1, Potri.018G134100.1, Potri.008G056400.1, Potri.016G094300.1, Potri.008G148000.1, Potri.007G062700.1
RLCKVIII	1	Potri.017G036300.1, Potri.007G124000.1, Potri.014G114200.1
RLPK	1	Potri.001G234200.1, Potri.010G213200.1, Potri.001G405500.1
*S*-domain Kinase	1	Potri.001G385200.1, Potri.001G438400.1, Potri.011G112000.1
ST-PK	5	Potri.012G057300.1, Potri.015G055200.1

*Class 1, transmembrane receptor kinase and related non-transmembrane kinases; Class 2, ATN1/CTR1/EDR1/GmPK6 like kinase; Class 3, casein kinase I; Class 4, non-transmembrane protein kinases; Class 5, other and unclassified protein kinase.*

#### Stress Related Proteins

In addition to the kinase superfamily proteins, several proteins related to abiotic and biotic stress responses were also identified as being *S*-acylated (Supplementary Table 1). In plants, proteins containing the NB-ARC domain are known to be involved in pathogen recognition and induction of plant defense responses (Ooijen et al., 2008). Five NB-ARC proteins were identified in this study to be *S*-acylated (Supplementary Table 1). *S*-acylation and myristoylation have been hypothesized to direct NB-ARC proteins to specific locations within the membrane (Bernoux et al., 2011). A few CBLs, which function as calcium sensors within the cell, were also identified in this work (Supplementary Table 1). CBLs play a critical role in abiotic stress response and are also involved in signaling pathways throughout growth and development (Batistic and Kudla, 2009). In *Arabidopsis*, the localization of three CBLs is known to be regulated by *S*-acylation (Batistic et al., 2008, 2012; Zhou et al., 2013). The plant PP2Cs, which interact with several PKs and regulate various signal transduction pathways (Rodriguez, 1998) were also identified in our study (Supplementary Table 1). Some of the PP2Cs are stress-induced and contain a putative *N*-myristoylation site. A conserved cysteine residue, which is also the potential site of *S*-acylation, is present close to the N-terminal end of PP2C. Other proteins in this category including RIN4 (Kim et al., 2005), ERD4, BON2, and redox enzymes such as thioredoxin, glutaredoxin, and glutathione peroxidase were also found to be *S*-acylated (Supplementary Table 1). Remorin, a lipid raft marker and stress-related protein, was also found to be *S*-acylated.

#### Proteins Related to Intracellular Trafficking

**FIGURE 6:**
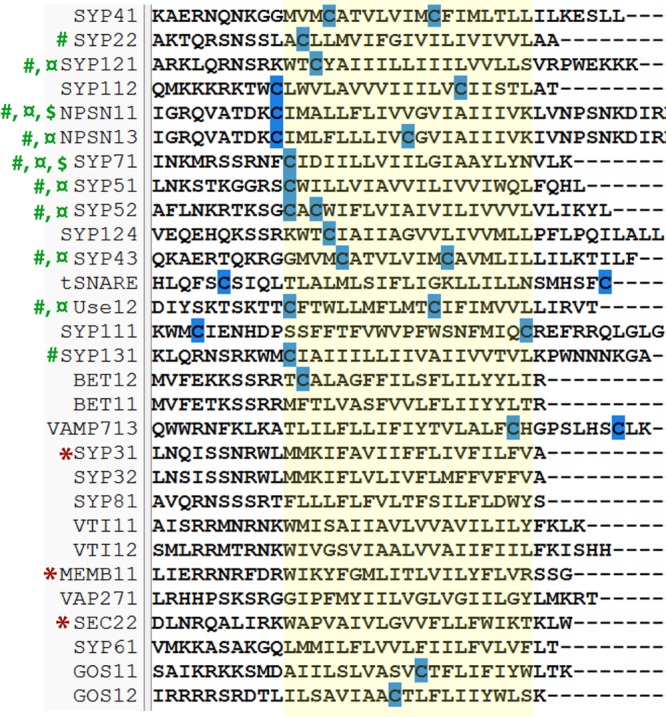
**Multiple alignment of poplar SNAREs.** The transmembrane domain is shaded in yellow and the conserved cysteine residues are highlighted in blue. #, poplar SNAREs identified as *S*-acylated; d’, the closest *Arabidopsis* homologs to these proteins have been identified as *S*-acylated by mass spectrometry; $, the closest *Arabidopsis* homologs to these proteins have been confirmed as *S*-acylated by *S*-acylation assay; *, the closest *Arabidopsis* homologs to these proteins have been confirmed to lack *S*-acylation by *S*-acylation assay (Hemsley et al., 2013).

Several proteins associated with intracellular membrane trafficking were identified as *S*-acylated (Supplementary Table 1). There are 16 proteins belonging to the superfamily SNARE (**Figure 6**), band 7 family proteins, tetraspanins and ALA-interacting proteins (ALIS1). The SNAREs serves as fusion mediators between vesicular and target membranes (Lipka et al., 2007). In an effort to predict *S*-acylation sites, two studies used multiple alignments to compare yeast (Roth et al., 2006) and *Arabidopsis* (Hemsley et al., 2013) SNAREs proteins. The results from both studies suggest conserved cysteine(s) are close to the cytoplasmic face of the TMD. Using the same methods, we investigated poplar SNAREs and our results align with previous studies, again placing the conserved cysteine(s) near the cytoplasmic side of the TMD (**Figure 6**). In addition, *S*-acylation was confirmed in the closest *Arabidopsis* orthologs SYP71 and NPSN11 using specific assays (Hemsley et al., 2013). Furthermore, the same study indicated that the absence of the conserved cysteine residues prevents *S*-acylation in some SNAREs, as appears to be the case in poplar (**Figure 6**). Another group included in this category is the Tetraspanins (Supplementary Table 1). Tetraspanins form tetraspanin-enriched microdomains by interacting with each other or with other membrane proteins (Wang et al., 2012). Tetraspanins are known to be involved in cell–cell adhesion and fusion, ligand binding, intracellular trafficking during pathogenesis, development, and immune response (Wang et al., 2012). Although there are 17 tetraspanin genes in the *Arabidopsis* genome (Wang et al., 2012), tetraspanin 1 only has been characterized and shown to be involved in leaf and root patterning (Cnops et al., 2000, 2006). Tetraspanin 3 was identified in the *Arabidopsis* plasmodesmatal proteome, suggesting a role in cell–cell communication (Fernandez-Calvino et al., 2011). The identification of proteins related to intracellular trafficking in our study, suggests *S*-acylation plays a crucial role in this process.

#### Cell Wall Associated Proteins

Many cell wall related proteins were identified as *S*-acylated in our proteomic study (Supplementary Table 1). Among these, CESA and GSL proteins are associated with cellulose and callose deposition at the surface of PM, respectively (Enns et al., 2005; Festucci-Buselli et al., 2007). As these enzymes contain multiple TMDs, *S*-acylation may be involved in regulating their activity rather than assisting localization to the PM. During normal plant growth, cellulose is more abundant than callose, but callose deposition has been linked with cytokinesis, male gametophyte development, pollen development and fertility, plasmodesmata and various abiotic and biotic stress responses (Jacobs et al., 2003; Enns et al., 2005; Toller et al., 2008; Chen et al., 2009; Vaten et al., 2011). It has been shown that KOR1, a GH nine family protein, interacts with CESA and plays an important role for normal cellulose synthesis (Nicol et al., 1998; Vain et al., 2014). Interestingly, a few endo-1,4-β-D-glucanases belonging to GH family 9 were also identified here alongside additional UDP-Glycosyltransferases (Supplementary Table 1). Based on these observations, we speculate that *S*-acylation mediates the interaction between CESA and KOR1. However, additional work is required to investigate if such interactions exist in poplar cells. The identification of CesA1, CesA3, and KOR1 in our poplar samples is further validated by the fact that cell cultures predominately consist of primary cell walls and all three proteins are known to be associated with the primary cell wall cellulose synthase complex found in the PM (Vain et al., 2014).

### *S*-Acylated DRM-Enriched Proteins

Detergent-resistant microdomains are lateral lipid patches within the PM known to support and regulate specific PM-associated biological processes (Rietveld and Simons, 1998). Recently, we used a quantitative proteomics approach to determine the major functions of DRMs in poplar (Srivastava et al., 2013). Relative to the PM, a total of 80 proteins, primarily associated with three functional classes, were enriched in poplar DRMs (Srivastava et al., 2013). We were intrigued that 56 of the 80 DRM-enriched proteins were putatively *S*-acylated as determined by a bioinformatics prediction tool (Srivastava et al., 2013). The present work allows us to compare proteins experimentally identified as *S*-acylated with the list of DRM-enriched proteins from our earlier work (Srivastava et al., 2013). Twenty-four of the 80 DRM-enriched proteins were experimentally found to be *S*-acylated (**Table 2** and Supplementary Table 1). Of these 24 proteins, 21 also contained predicted *S*-acylation sites (**Table 2**). The localization of a large proportion of these proteins to the PM or more specifically to DRMs is known. Two DGKs were identified as *S*-acylated proteins in our study despite having no predicted *S*-acylation sites. DGKs are PM localized proteins known to convert diacylglycerol to phosphatidic acid, both of which act as secondary messengers (Testerink and Munnik, 2005). Sequence analysis found few conserved cysteine residues which represent potential *S*-acylation sites in these two DGKs. The *Arabidopsis* homolog of poplar DGK3 was also found as being *S*-acylated by proteomics (Hemsley et al., 2013). The experimental confirmation of *S*-acylation in some DRM-enriched proteins suggests that this PTM may play a role in localizing proteins to membrane microdomains rich in sphingolipids, sterols, and phospholipids that carry saturated fatty acids. Supporting this hypothesis is the example of *Arabidopsis* ROP6 whose transient *S*-acylation upon GTP binding induces its partitioning into DRMs (Sorek et al., 2007).

**Table 2:** *S*-acylated proteins enriched in poplar detergent-resistant microdomains (DRM).

Potri.008G082100.1	ALA-interacting subunit 1, ALIS1
Potri.012G131300.1	Amino acid transporter 1
Potri.004G182800.1	BR-signaling kinase 1
Potri.005G234900.1	Calcium-binding tetratricopeptide protein
Potri.012G108200.1	Calcium-dependent phospholipid-binding family protein
Potri.011G003400.1	Calcium Dependent Protein Kinase 21
Potri.009G052700.1	Calcium Dependent Protein Kinase 7
Potri.018G096700.1	Diacylglycerol Kinase 3
Potri.006G174700.1	Diacylglycerol Kinase 7
Potri.001G012000.1	Glucan Synthase like-10
Potri.001G011900.1	Glucan Synthase like-10
Potri.015G089300.1	Glucan Synthase like-8
Potri.012G070500.1	Band 7 family protein
Potri.017G078100.1	Band 7 family protein
Potri.018G074300.1	Leucine Rich Repeat Protein Kinase
Potri.016G126300.1	Leucine Rich Repeat Protein Kinase
Potri.007G065000.1	Leucine-rich repeat family protein
Potri.006G081400.1	Protein phosphatase 2C
Potri.015G073000.1	ROP10
Potri.012G044600.1	Leucine Rich Repeat Protein Kinase
Potri.002G157700.1	Remorin
Potri.014G081300.1	Remorin
Potri.016G088200.1	SNARE/SYP71
Potri.009G015100.1	Tetraspanin 3

## Conclusion

Protein *S*-acylation plays an important role in plant growth and development by regulating the function of proteins associated with diverse biological processes. In this study, we have identified some known (e.g., remorin, SNAREs, CBLs, CDPKs, RIN4, PATs), as well as several novel *S*-acylated proteins in poplar. Also identified as *S*-acylated in our study are many unknown proteins and proteins that contain DUF (Supplementary Table 1). Although more experimental data are needed to confirm the site of *S*-acylation for these proteins, our work pave the way toward a better understanding of the role of *S*-acylation in higher plants. Proteins found to be *S*-acylated in both *Arabidopsis* and poplar may also share a common means of regulation by *S*-acylation. In addition to improving our knowledge of the various functions of *S*-acylation in plants, the data from this study can also be used to predict *S*-acylation in related proteins in other plant species.

## Author Contributions

VS and VB conceived the study. JW performed experimental work with input from VS and BF. VS and EM analyzed the data. VS and VB wrote the manuscript.

## Conflict of Interest Statement

The authors declare that the research was conducted in the absence of any commercial or financial relationships that could be construed as a potential conflict of interest.

## Footnotes

1 http://bioinfo.bti.cornell.edu/cgi-bin/itak/index.cgi

2 http://taxonomy.zoology.gla.ac.uk/rod/treeview.html

3 http://bioinfo.bti.cornell.edu/cgi-bin/itak/index.cgi

## Abbreviations

ABE: acyl-biotin exchange
CBL: calcineurin B-like protein
CDPK: calcium-dependent protein kinase
CesA: cellulose synthase
DGK: diacylglycerol kinase
DHHC-CRD: aspartate-histidine-histidine-cysteine cysteine-rich domains
DRM: detergent-resistant microdomains
DUF: domains of unknown function
GH: glycoside hydrolase
GSL: glucan synthase-like
KOR1: Korrigan
LRR-PK: leucine-rich repeat protein kinase
LRR-RLK: leucine-rich repeat-receptor like kinases
PAT: palmitoyl acyltransferases
PTM: post-translational modification
RLCK: receptor-like cytoplasmic kinases
SNAREs: soluble NSF attachment protein receptor
TMD: transmembrane domain
