# Supplementary material for: Proteomic Analysis of a Poplar Cell Suspension Culture Suggests a Major Role of Protein S-Acylation in Diverse Cellular Processes
Source: Front Plant Sci. 2016 Apr 12;7:477. doi: 10.3389/fpls.2016.00477 (PMC4828459; doi:10.3389/fpls.2016.00477)
Supplement: Supplementary file 1 [file Data_Sheet_1.DOCX]

Proteomic analysis of a poplar cell suspension culture suggests a major role of protein S-acylation in diverse cellular processes

Vaibhav Srivastava, Joseph Weber, Erik Malm, Bruce W. Fouke & Vincent Bulone


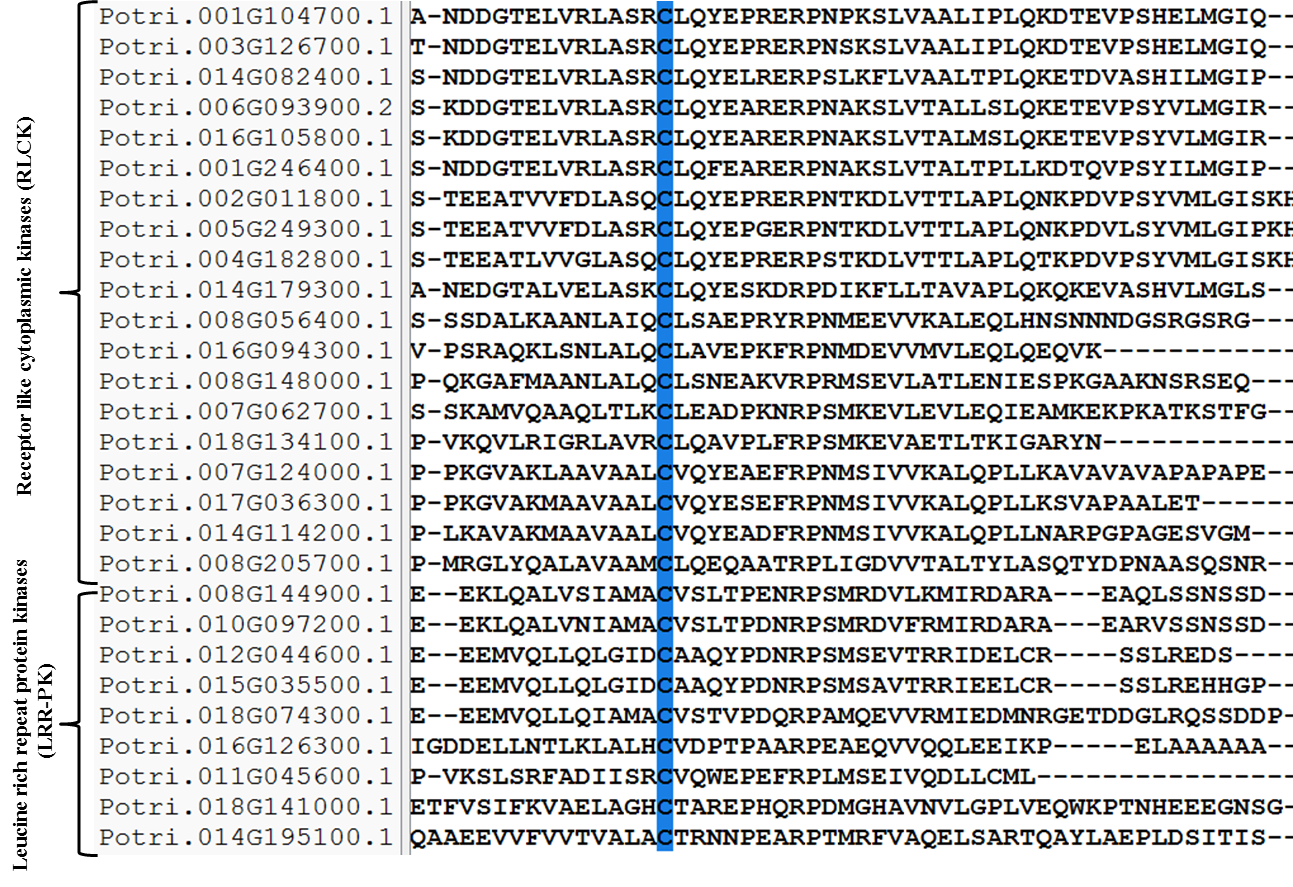


**Supplemental Figure 1:** Multiple alignment of poplar leucine rich repeat protein kinases (LRR-PK) and receptor like cytoplasmic kinases (RLCK) identified as S-acylated. The conserved cysteine residue is highlighted in blue.
